# Bench-Press Performed With a Velocity- and Tempo-Based Approach: Are There Differences in Volume Load, Time Under Tension, and Metabolic Demands?

**DOI:** 10.1177/19417381261416535

**Published:** 2026-02-11

**Authors:** Afonso Fitas, Sergio Miras-Moreno, João Henriques Oliveira, Margarida Cidrais, Pedro Pezarat-Correia, Brad J. Schoenfeld, Goncalo V. Mendonca

**Affiliations:** † Neuromuscular Research Laboratory, Faculdade de Motricidade Humana, Universidade de Lisboa, Cruz Quebrada, Dafundo, Portugal; ‡ CIPER, Faculdade de Motricidade Humana, Universidade de Lisboa, Cruz Quebrada, Dafundo, Portugal; § Department of Physical and Sports Education, Faculty of Sport Sciences, Sport and Health University Research Institute (iMUDS), University of Granada, Granada, Spain; ‖ Egas Moniz School of Health and Science, Portugal; ¶ Department of Exercise Science and Recreation, CUNY Lehman College, Bronx, New York

**Keywords:** energy, exercise, fatigue, oxygen uptake, resistance training

## Abstract

**Background:**

Velocity-based training (VBT) is a resistance training approach that uses lifting velocity to determine training load and track strength progress. This study determined the impact of a VBT versus a tempo-based training (TBT) approach on volume load and time under tension during a single set of submaximal bench press performed to failure.

**Hypothesis:**

VBT would result in larger volume load and similar time under tension as TBT.

**Study Design:**

Randomized-crossover design.

**Level of Evidence:**

Level 3.

**Methods:**

A total of 14 healthy men (24.1 ± 5.8 years) performed free-weight bench-press exercise at low intensities (12%, 16%, 20%, and 24% of 1-repetition maximum [1RM]) with oxygen uptake ( $\dot{V}$ O_2_) measurements. $\dot{V}$ O_2_ was then extrapolated to a set performed at 70% 1RM to failure and the accumulated O_2_ deficit was calculated together with the relative energy contribution of aerobic and anaerobic metabolism. Mechanical data were collected with a linear encoder.

**Results:**

Despite the lack of differences between conditions for total time under tension (*P* > 0.05), VBT achieved a higher volume load at set failure (*P* < 0.05). Moreover, the VBT condition resulted in a larger total $\dot{V}$ O_2_ from set initiation to termination (*P* < 0.01). Conversely, the accumulated O_2_ deficit did not differ between conditions (*P* > 0.05). Compared with TBT, VBT elicited a higher relative contribution of aerobic energy (VBT, ~41%; TBT, 33%) and a lower relative contribution of anaerobic energy (VBT, ~59; TBT, 67%) during exercise (*P* < 0.01).

**Conclusion:**

VBT is an effective strategy to enhance volume load during bench-press performed to failure at 70% 1RM. This effect occurs without compromising time under tension. These findings are associated with a higher contribution of aerobic energy supply to exercise.

**Clinical Relevance:**

VBT may be beneficial for athletes aiming to maximize volume load in response to resistance exercise.

Velocity based training (VBT) is a resistance training approach that uses lifting velocity to determine training load and track strength progress.^9^ Conceivably, this enables a recursive fine-tuning of the resistance training stimulus towards functional overreaching, while avoiding excessive loading and effort that may be conducive to maladaptation.^18^ When using VBT, trainees are required to produce maximal intended velocity during the concentric phase of a given exercise.

Despite its broad spectrum of applications, VBT has been used most frequently for inducing adaptations in muscle strength and power. There are additional features to consider when designing VBT interventions aimed at developing muscle strength and power. For instance, the termination of each set is typically dependent on velocity loss defined as a percentage threshold (e.g., loss of 10% or 20% of mean concentric velocity) or an absolute drop-off (e.g., avoid going below a mean concentric velocity of 0.5 m s^–1^).^29^ This is relevant because a lower velocity loss seems to enhance strength and power adaptations, while a higher velocity loss promotes hypertrophy, probably due to the lesser amount of training volume and fatigue inherent to each percentage velocity loss.^14^

While much research has been conducted to examine the impact of VBT on muscle strength and power, information remains scarce on how it effects muscle hypertrophy. There is compelling evidence that the hypertrophic response to VBT (interventions lasting 6 to 8 weeks) can be enhanced by prescribing a larger percentage velocity loss in each set (between 40% and 50%).^14^ Since it is well-established that the magnitude of muscle hypertrophy depends on the accumulated training volume and the proximity to volitional failure at the termination of each set, these findings are not surprising.^3,22,24^ However, an issue that remains unresolved is whether VBT can potentiate increases in muscle size beyond those seen with a more conventional approach to muscle hypertrophy (i.e., based on a preset movement tempo for concentric and eccentric phases).

According to a recent narrative review focusing on the interaction between movement tempo and muscle hypertrophy, muscle hypertrophy can be potentiated by the combination of slower movement in the eccentric phase with a faster movement during the concentric phase (which, essentially, corresponds to the VBT approach).^32^ Specifically, some evidence suggests that a fast movement velocity may generate a higher level of maximal muscle force compared with that seen during slow tempo movements.^8,12^ Similar observations were made concerning the number of repetitions. Past studies have consistently shown that faster movement velocity enables performing more repetitions to failure at a given relative load, which translates into a larger volume load (absolute load multiplied by the number of repetitions and the number of sets) in response to exercise.^23,30,31^ These findings raise the possibility that VBT might be an effective stimulus to increase muscle growth.

There are at least 2 aspects that may affect the relationship between VBT and muscle hypertrophy. First, in all of the above-mentioned studies, the tempo of each lift was varied by increasing the velocity of both phases of movement (concentric and eccentric). Second, time under tension, another determinant of volume, was reduced with faster movement tempos (despite an increased number of repetitions). This is relevant because a greater time under tension has been shown to produce larger increases in rates of muscle protein synthesis and this may help promoting muscular hypertrophy.^4^ Again, considering that the eccentric phase of each lift should be controlled and relatively slow on a repetition-by-repetition basis, this might also be different with VBT. However, whether exercising to failure with a VBT approach is compatible with an increased volume load without compromising total time under tension (compared with a conventional hypertrophic tempo-based [TBT] exercise prescription), is presently unknown. Hypothetically, if this were to occur, the metabolic demands of VBT would necessarily differ from those inherent to TBT exercise prescription.

The present study aimed to examine the impact of VBT versus TBT on volume load and time under tension in response to a single set of submaximal bench press performed to failure. In addition, we sought to determine whether both exercise approaches resulted in dissimilar acute metabolic demands. We hypothesized that the VBT approach would result in a larger volume load and similar time under tension as TBT. Finally, considering the finite nature of the accumulated oxygen deficit in response to exercise performed to failure (limited amount of work that can be performed anaerobically before reaching exhaustion), it was also hypothesized that the larger volume load achieved with VBT would rely on a greater contribution of aerobic energy at set termination.

## Methods

### Participants

Based on pilot data from 5 participants with past experience in the free-weight bench press (other than those included in this study, but with a relative bench-press 1 repetition maximum [1RM] of 1.1 ± 0.1), we determined that the impact of performing the concentric phase of each repetition to failure at maximum velocity on the relative contribution of aerobic and anaerobic energy (versus a cadenced approach) is characterized by a partial eta-squared of 0.51. Considering an alpha level of 0.05, a sample size of 11 participants was estimated to achieve 80% of power of correctly rejecting the null hypothesis (1-way analysis of variance [ANOVA] repeated measures, within factors: 1 group and 2 conditions) (G*power software, Version 3.1.9.7). To ensure sufficient statistical power for detecting eventual differences between conditions (VBT versus TBT approach), we opted to recruit a total of 14 participants (aged 18 to 34 years old) with previous experience in the free-weight bench-press exercise, but not necessarily involved in a consistent resistance training regimen at time of study entry (Table 1).

**Table 1.** Descriptive characteristics of participants

Variable	N = 14 male participants
Age, years	24.1 ± 5.8
Height, cm	179.2 ± 8.1
Body mass, kg	75.4 ± 11.2
Bench-press 1RM, kg	81.1 ± 21.5
Relative bench-press, 1RM	1.1 ± 0.2
Resting $\dot{V}$ O_2_, ml kg^–1^ min^–1^	6.1 ± 0.8
Estimated $\dot{V}$ O_2_ at 70% 1RM, ml kg^–1^ min^–1^	31.1 ± 7.7

Values are mean ± SD. 1RM, 1-repetition maximum; $\dot{V}$ O_2_, oxygen uptake.

The study design was explained carefully to each participant during the first visit. Participants were then required to provide written informed consent for the experimental procedures, which complied with the Declaration of Helsinki. The study received ethical approval from the Institutional Ethics Committee (CEFMH no. 28/2023). The inclusion criteria were as follows: (1) absence of cardiovascular, respiratory, metabolic, and orthopaedic disease; (2) no musculoskeletal injury during the past 6 months; (3) individual experience in performing the free-weight bench-press exercise with the proper technique^11^; (4) self-reported to be free from taking exogenous anabolic-androgenic steroids or other drugs or substances expected to affect physical performance or hormonal balance for several months before or during this study; and (5) not taking any medication. Participants were accustomed to performing bench press full-depth repetitions to failure (focusing either on velocity or cadence); thus, extensive familiarization with exercise technique was not required.

Each participant was instructed to avoid heavy exercise for at least 72 h before testing and to abstain from eating from midnight until the testing session on the subsequent morning (fasting conditions). Participants were also asked to refrain from caffeine ingestion for 24 h and to empty their bladders immediately before testing. The participants’ risk of negative outcomes was minimized as much as possible and no withdrawals were noted during the course of the study.

### Study Design

We employed a randomized-crossover design to investigate the research question. All participants were requested to visit the laboratory on 5 nonconsecutive occasions with a minimum of 72 h between sessions (maximum of 120 h). Testing sessions were performed during the morning, between 0800 hours and 1100 hours, in a laboratory setting at a temperature between 22°C and 24°C and a relative humidity of between 44% and 56%. The first session was structured to obtain the individual free-weight bench press 1RM. The second session involved measuring oxygen uptake ( $\dot{V}$ O_2_) in response to single sets performed to failure at 12% and 20% 1RM. The third session followed a similar approach, but using submaximal loads of 16% and 24% 1RM. During the fourth and fifth sessions, all participants completed a single set of bench-press repetitions to failure at 70% 1RM with $\dot{V}$ O_2_ and linear velocity measurements (2 conditions: VBT or TBT). This relative load was chosen because previous research indicates that, for the bench press, the differences in concentric velocity between conditions focusing on maximum velocity or moderate tempo (~20 repetitions per minute) become too small beyond 70% 1RM.^23^ The beginning of each lift was always spotted by a member of the research team. The bar was lifted from the rack columns with the help of the spotter and handed to the participant only after receiving verbal confirmation of safe handling. The end point of all successful lifts was defined as reaching a concentric full-elbow extension coming from a full-depth eccentric phase (i.e., bar touching the chest at the nipple line to ensure full range of motion). All repetitions were performed without bouncing the barbell against the chest, without intentionally pausing at the transition between the eccentric to concentric phases, and without raising the lower back off the bench.

### Bench-Press 1RM

Before 1RM testing, each participant’s body mass was measured on a digital scale to the nearest 0.01 kg (TANITA BF-350 body composition analyzer) and height was taken with a stadiometer to the nearest 0.5 cm (Secca 216) without shoes and wearing lightweight clothes.

The bench-press exercise was performed using a flat-weight bench and an Olympic bar (unloaded bar mass, 20 kg) (BOXPT). Emphasis was placed on completing each lift at full depth, using the prescribed technique. Participants assumed the supine position and all attempts were completed using 5 points of body contact (i.e., the firm planting of their feet, gluteus maximus, back, and head), beginning with their elbows extended and gripping the bar aligned with the individual nipple line.^5^ The grip width was defined as the distance measured between the elbows at 90° shoulder abduction and was kept consistent throughout all testing sessions. Participants were given a countdown before beginning each attempt, and specific verbal encouragement was provided throughout each lift.

At the beginning of testing, all participants completed a warm-up protocol consisting of 10 bench-press repetitions (unloaded bar). The concentric phase of warm-up repetitions varied between maximal and tempo intended velocity throughout the full range of motion (5 repetitions at maximal velocity, and another 5 at 1.5 seconds per repetition). Assessments of 1RM consisted of 5 sets at an estimated 20% (3 repetitions), 40% (3 repetitions), 60% (3 repetitions), 80% (1 repetition), and 90% (1 repetition), followed by the first 1RM attempt.^7^ A maximum of 5 1RM attempts were allowed with 3 minutes passive recovery between sets. The load was increased 0.5 kg to 2.5 kg after successful 1RM attempts until no further weight could be lifted with proper technique. Participants were prohibited from wearing supplemental equipment (e.g., elbow sleeves).

### Oxygen Demand in Response to Submaximal Sets Performed to Failure

The second and third testing sessions were designed to measure bench-press $\dot{V}$ O_2_ during steady-state conditions and, from these values, extrapolate the $\dot{V}$ O_2_ of this specific exercise when lifting 70% of 1RM. The bench-press sets were performed to failure at 12% and 20% 1RM (session 2) and at 16% and 24% 1RM (session 3). These relative loads were chosen based on previous research showing that they are sufficiently heavy to induce $\dot{V}$ O_2_ values above resting, but light enough to be performed every ~3 minutes to 5 minutes.^21,28^ Depending on the absolute load to be lifted by the participants at each relative intensity, a 3-kg or 8-kg bar was used in combination with plates varying from 0.5 kg to 5 kg (Technogym). In each session, load presentation was counterbalanced between participants to avoid a series effect (i.e., the order of presentation was inverted successively between participants). $\dot{V}$ O_2_ was measured continuously in the seated position for 10 minutes before exercise. In both testing sessions, exercise bouts were interspersed with a seated recovery period long enough to allow $\dot{V}$ O_2_ to return to baseline (within 2 ml kg^–1^ min^–1^ of resting $\dot{V}$ O_2_). No warm-up was allowed before exercise and the bench-press tempo was set at 20 repetitions per minute by an electronic metronome sound (eccentric, 1.5 seconds; pause, 0 seconds; concentric, 1.5 seconds; pause, 0 seconds).^21^

### Relative Contribution of Aerobic and Anaerobic Energy in Response to Submaximal Sets Performed to Failure

During the fourth and fifth sessions, the participants began by completing a warm up similar to that described for 1RM testing. Then, as described for session 1 and session 2, the participants were allowed to rest in the seated position for 10 minutes before exercise. Finally, they were tested in response to the bench press performed to failure at 70% 1RM in 2 different conditions (VBT and TBT). These conditions were also presented in a counterbalanced fashion between participants. The eccentric phase of all lifts was paced at 1.5 seconds in both conditions, and no pause was allowed during transition to the concentric phase. For the VBT condition, each participant was instructed to produce maximal intended concentric velocity (MAX) for all repetitions up to failure (eccentric, 1.5 seconds; pause, 0 seconds; concentric, MAX; pause, 0 seconds). Velocity feedback was provided verbally after completing each repetition to encourage all participants to perform all lifts at MAX.^13^ Conversely, for the TBT condition, the concentric phase of each lift was paced at 1.5 seconds and the participants were constantly encouraged to respect the pace of the metronome during the completion of each repetition (eccentric, 1.5 seconds; pause, 0 seconds; concentric, 1.5 seconds; pause, 0 seconds). This tempo was chosen because past research has shown that the impact of bench-press velocity on the number of repetitions completed at a given relative load tends to largely dissipate at faster tempos (specifically at 70% 1RM),^23^ As fatigue accumulated, participants became progressively unable to maintain the prescribed tempo. Despite this, all participants were further encouraged to pace themselves by keeping the metronomic pace as best as possible until failure (i.e., not reaching a concentric full-elbow extension coming from a full-depth eccentric phase). At set failure, 1 spotter assisted each participant by re-racking the bar back. Each participant’s $\dot{V}$ O_2_ was measured continuously from the beginning to the end of each set.

Expired gas measurements were taken using a portable mixing chamber (Metamax I, Cortex), which was calibrated before each test with a known volume and with known gas concentrations. Measurements were performed with a previously validated linear position transducer, which sampled the bar’s velocity at a frequency of 1000 Hz (Chronojump).^20^ The tether of the linear position transducer was attached to the barbell using a Velcro strap and remained perpendicular to the ground during testing.

### Data Analysis

For data obtained during session 2 and session 3, the mean of the last 2 minutes of the 10-minute seated rest was taken as representative of the participant’s $\dot{V}$ O_2_. To standardize the time window of analysis between the different relative loads tested in these submaximal sessions, and to ensure that extrapolations were made using steady-state data, all sets with a duration of <210 seconds were excluded. Steady-state $\dot{V}$ O_2_ was taken as the mean value obtained between 180 seconds and 210 seconds in all sets that fulfilled the above-defined criterion.

The accumulated oxygen difference (AOD) obtained in sessions 4 and session 5 was calculated by subtracting actual cumulative $\dot{V}$ O_2_ from the estimated O_2_ demand in both conditions,^16^ and was not adjusted for the contribution of stored oxygen. The AOD value was expressed relative to body mass (ml kg^–1^). The relative contribution of anaerobic and aerobic energy during exercise was then determined by the AOD and the actual cumulative $\dot{V}$ O_2_, respectively. Based on the available literature, there is some evidence that the inclusion of resting $\dot{V}$ O_2_ in the individual regression models (as *y* intercepts) increases the robustness of the relationship between external and internal load (especially when there are few submaximal bouts for AOD computation).^19^ For this reason, the AOD (as well as the relative aerobic and anaerobic energy contribution) of all participants was recalculated and compared with that obtained without setting the *y* intercept to the participants’ resting $\dot{V}$ O_2_.

Mean values of concentric velocity, power and displacement of the bar were taken from the start of the concentric phase of each lift to the instant corresponding to the maximum height of the bar. The duration of the eccentric phase of each lift was taken from the start of the eccentric phase of each lift to the instant corresponding to the lower height of the bar. Finally, total time under tension was calculated individually as the cumulative value of time spent under tension in each set (Σ concentric time and eccentric time of all set repetitions).

### Statistical Analysis

Descriptive data are presented as means and standard deviations. Data were assessed for normality, sphericity, and homoscedasticity using the Shapiro-Wilk, Mauchly’s, and Levene’s tests, respectively. One-way repeated measures ANOVAs were computed to examine differences between conditions (VBT versus TBT approach) on all dependent variables.

Because a VBT approach with verbal augmented feedback can have an ergogenic effect on bench-press performance,^13^ we also explored whether total volume load (i.e., number of sets × number of repetitions × load lifted) differed between conditions at set failure. We obtained a condition main effect for volume load and for the relative contribution of aerobic energy during exercise. For this reason, we repeated the statistical analysis on volume load between conditions using the delta in relative aerobic contribution between conditions as a covariate (analysis of covariance [ANCOVA]). This was done with the purpose of exploring whether the differences between conditions in volume load might be associated with a dissimilar relative contribution of aerobic energy during exercise. Effect sizes (ES) were calculated using the partial eta-squared values obtained for each variable (corresponding to a small, medium and large effect of 0.01, 0.06, and 0.14, respectively).^6^ Statistical analyses were performed using SPSS Version 27.0 (SPSS, Inc) and statistical significance was set a priori at *P* < 0.05.

## Results

**Figure 1.**
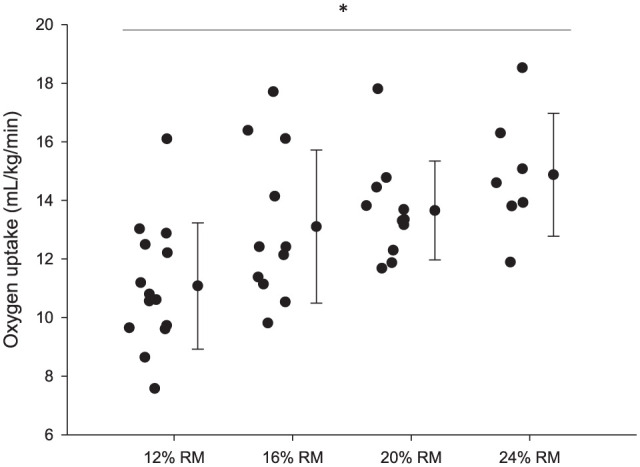
Oxygen uptake obtained at steady-state conditions in response to bench press sets performed to failure at different relative loads (12%, 16%, 20%, and 24% of 1RM). Nonstationary data points (bench-press failure in <180 seconds) were excluded from the analyses and are not shown in the figure. 1RM, 1-repetition maximum *Relative load main effect (*F* = 224.4; *P* < 0.001; ES, 0.95).

Table 1 shows the participants’ demographic characteristics, bench-press 1RM and steady-state $\dot{V}$ O_2_ values (obtained at rest and estimated for the bench press at 70% 1RM). The relative strength of this group of participants was ~1.1, which confirms their recreationally trained background at study entry. Figure 1 depicts the steady-state $\dot{V}$ O_2_ at each relative load. There was a relative-load main effect (*F* = 224.4; *P* < 0.001; ES, 0.95), showing that $\dot{V}$ O_2_ increased statistically as function of relative load. The figure also shows the individual data points that were compatible with steady-state $\dot{V}$ O_2_ at each relative load. As can be seen, there was a progressive loss of data points as relative load increased from 12% to 24% 1RM. This means that the number of participants that were able to reach at least 210 seconds of continued exercise before failure decreased as relative load increased.

As shown in Table 2, the VBT condition resulted in a larger cumulative O_2_ response (total oxygen uptake from set initiation to termination) in response to the bench press exercise performed to failure at 70% 1RM (*F* = 9.1; *P* < 0.01; ES, 0.41). In contrast, neither the AOD (F = 0.01; *P* = 0.89; ES, 0.001) nor the total estimated $\dot{V}$ O_2_ demand (*F* = 1.6; *P* = 0.24; ES, 0.10) differed between conditions. The values obtained after recalculating both these variables with the inclusion of resting $\dot{V}$ O_2_ into the individual regression models (as *y* intercepts) did not differ from those presented in Table 2 (data not shown). In contrast to that observed for the cumulative $\dot{V}$ O_2_ and the AOD, the relative contribution of each energy system for the bench-press exercise differed statistically between conditions. Specifically, compared with that seen with TBT, the VBT condition elicited a higher relative contribution of aerobic energy and a lower relative contribution of anaerobic energy during exercise (*F* = 14.3; *P* = 0.002; ES, 0.52 for both) (Table 2).

**Table 2.** Accumulated oxygen uptake and deficit in response to a single set performed to failure at 70% 1RM with VBT and TBT approaches

Variable	VBT	TBT	*P* value
Total estimated $\dot{V}$ V.O_2_ demand, ml kg^–1^	31.4 ± 14.3 (23.1-39.7)	28.6 ± 10.8 (22.4-34.8)	0.23
Cumulative $\dot{V}$ V.O_2_, ml kg^–1^	11.9 ± 4.9 (9.1-14.7)	8.9 ± 2.9 (7.2-10.5)	0.01
Accumulated oxygen deficit, ml kg^–1^	19.5 ± 10.8 (13.2-25.8)	19.7 ± 8.8 (14.6-24.7)	0.90
Aerobic, %	40.9 ± 14.7 (32.5-49.5)	32.7 ± 9.5 (27.2-38.2)	0.002
Anaerobic, %	59.1 ± 13.3 (50.5-67.5)	67.3 ± 8.9 (61.8-72.8)	0.002

Values are mean ± SD (95% CI). 1RM, 1-repetition maximum; VBT, velocity-based training; V. $\dot{V}$ O_2_, oxygen uptake; TBT, tempo-based training.

Despite the lack of differences between conditions for total time under tension (*F* = 0.06; *P* = 0.81; ES, 0.01), duration of the eccentric phase (*F* = 0.12; *P* = 0.73; ES, 0.02) and concentric displacement of the bar (*F* = 1.49; *P* = 0.24; ES, 0.10), VBT enabled the participants to complete more repetitions (*F* = 8.9; *P* = 0.01; ES, 0.41) and reaching a higher volume load at set failure (*F* = 7.2, *P* = 0.02; ES, 0.37, respectively) (Table 3). Mean concentric power (*F* = 31.4; *P* < 0.001; ES, 0.71) and velocity (*F* = 23.8; *P* < 0.001; ES, 0.64) generated during the bench press was statistically higher in the VBT conditions.

**Table 3.** Mechanical parameters in response to a single set performed to failure at 70% 1RM with VBT and TBT approaches

Variable	VBT	TBT	*P* value
Number of repetitions	14.4 ± 2.9 (12.7-16.1)	12.7 ± 2.5 (11.3-14.3)	0.01
Volume load, kg	822.5 ± 301.5 (648.4-996.6)	733.2 ± 278.0 (572.7-893.7)	0.02
Mean concentric velocity, m s^–1^	0.39 ± 0.08 (0.35-0.44)	0.31 ± 0.05 (0.28-0.34)	<0.001
Mean concentric power, W	212.3 ± 51.5 (182.6-242.0)	180.1 ± 58.6 (146.3-213.9)	<0.001
Bar-concentric displacement, cm	40.7 ± 5.1 (37.8-43.8)	39.5 ± 5.0 (36.6-42.4)	0.24
Duration of the eccentric phase, sec	1.23 ± 0.21 (1.06-1.37)	1.26 ± 0.11 (1.17-1.37)	0.73
Total time under tension, sec	31.9 ± 6.3 (27.1-36.7)	31.3 ± 6.8 (26.1-36.6)	0.81

Values are mean ± SD (95% CI). 1RM, 1-repetition maximum; VBT, velocity-based training; TBT, tempo-based training.

Finally, as the participants were able to accumulate a larger volume load in the VBT condition and this was accompanied by an increased relative contribution of aerobic energy during exercise, we repeated the ANOVA on volume load, controlling for the effects of delta in relative aerobic contribution between conditions as a covariate. The results from this ANCOVA showed that, after controlling for the effects of the delta increase in relative contribution of aerobic energy resulting from VBT, the differences in volume load between conditions were no longer statistically significant (*F* = 4.3; *P* = 0.06; ES, 0.26).

### Discussion

This study found that a larger volume load can be achieved during the bench press performed to failure with a VBT (1.5 second, 0 second, MAX, 0 second) versus a TBT approach (1.5 second, 0 second, 1.5 second, 0 second approach). In addition, it was also found that, despite being associated with a faster lifting cadence, a VBT approach does not compromise time under tension because more repetitions can be completed when benching to failure. Finally, we provided preliminary evidence that the additional volume load accumulated with a VBT approach partially relies on a higher relative contribution of the aerobic system to fuel the bench-press exercise.

To our knowledge, this is the first experimental design to compare the mechanical and metabolic profile of resistance exercise focusing on a VBT approach with that seen in response to a paradigm based on a preset movement tempo for concentric and eccentric phases (TBT approach). We observed that the VBT approach enables the completion of a greater number of bench press repetitions during a single set performed to failure, without compromising time under tension. Volume load is calculated as the product of the load and the number of repetitions. The calculation is an approximation of mechanical work (force × distance), with the assumption that all the repetitions are performed through the same range of motion.^25^ By showing that the concentric displacement of the bar did not differ between both conditions, we provide evidence that a greater amount of mechanical work was indeed attained when using the VBT approach. However, it is important to note that there is no universally accepted definition of exercise volume load. For instance, volume can also be calculated as the cumulative time that a muscle group is under tension or contraction during an exercise session. The relevance of the combination of both variables for exercise volume was shown in a previous study.^26^ Specifically, that study demonstrated that, for a given volume load, the magnitude of muscular fatigue varies as a function of time under tension. Similarly, for conditions involving a given time under tension, muscular fatigue varies as a function of volume load. Ultimately, this means that a potential difference in exercise volume can be accepted only in circumstances that enable the control of both volume load and time under tension, as was the case in the present study.

Previous research indicates that velocity alters the relationship between percentage 1RM and number of repetitions, with faster velocities producing a larger number of repetitions.^23,31^ However, these experimental designs did not control the eccentric phase of each lift, which means that the greater volume load achieved at faster tempos was likely associated with the positive effects of the stretch-shortening cycle on concentric performance. The strength of the relationship between eccentric cadence and concentric motor performance is known to be quite evident for the bench press exercise.^30^ Thus, it is likely that most of the additional work that can be done while benching at faster tempos depends on the combined effect of increased elastic energy reutilization and increased neural potentiation.^27^ In contrast, when controlling the eccentric phase of each lift at a steady cadence (as done in this study), this effect can no longer offer any explanation for the association of the VBT condition with heightened external work, increased concentric velocity or power output. According to the present findings, the mean duration of the eccentric phase was similar between both conditions and very close to the metronomic pace that was prescribed for the completion of the set (~1.5 s). Thus, the enhancement of work capacity observed when exercising to failure with a VBT approach was not likely caused by any further activation of the stretch-shortening cycle.

The AOD did not differ between conditions at exercise failure. It has been postulated that the AOD provides an estimation of anaerobic contribution to overall energy expenditure.^1^ However, there is some controversy concerning its validity, mostly because of the limitations inherent to the studies that focused on comparisons with metabolic measurements of anaerobic adenosine triphosphate (ATP) production.^2,10,17^ The main limitation with the metabolic measurements of anaerobic ATP production from muscle biopsy data is that the active muscle mass is unknown, which precludes accurate assessment as to the validity of the AOD method.^19^ Despite this, there is some consensus that the validity of the AOD to determine anaerobic capacity is improved when applied to exercises involving a smaller amount of muscle mass (i.e., more localized exercise).^19^ The lack of differences between conditions for the AOD strongly indicate that the anaerobic ATP production was of similar magnitude when benching to failure with a VBT and a TBT approach. It is relevant to note that the AOD obtained in the present study is comparable with that previously reported for the bench press performed to failure at 80% 1RM (~19 ml kg^–1^).^28^ Taken together, this provides some evidence that, when benching to failure, the absolute contribution of anaerobic energy for exercise remains roughly the same even when using different lifting approaches or relative loads. Importantly, given that the AOD was not different between conditions, we provide preliminary evidence that the additional volume load accumulated during exercise with the VBT approach cannot be explained by a heightened contribution of anaerobic energy for work output in absolute terms.

As opposed to that seen for the AOD, the cumulative $\dot{V}$ O_2_ of benching to failure at 70% 1RM was enhanced with the VBT approach. In contrast with the AOD, which represents the portion of energy derived from the anaerobic metabolism, the cumulative $\dot{V}$ O_2_ reflects the contribution of aerobic energy to exercise.^16^ The sum of both components equals the equivalent of total energy expenditure quantified in terms of $\dot{V}$ O_2_. Based on the determination of these 3 parameters, it is possible to compute the proportion of aerobic and anaerobic energy for a given motor task. The present data indicate that the VBT approach shifted the proportion of energy contribution towards an aerobic predominance. Thus, it can be said that a larger contribution of aerobic energy provides a partial explanation for the additional volume load that was accumulated when benching with the VBT condition. This notion is somewhat corroborated by our findings showing that such differences in volume load between conditions were no longer statistically significant after controlling for the effects of enhanced aerobic contribution that accompanied the VBT approach. However, this conclusion should be interpreted cautiously as the *P* value (0.06) raises uncertainty as to probability of a differential effect.

### Limitations

There are at least 3 important limitations to this study. First, it is unknown whether these findings transfer to other free-weight exercises. Second, our sample included male participants that were not required to have been formally participating in resistance training at study entry (but were accustomed to the bench-press exercise). It remains unclear whether these findings would also be sustained for female participants or people with more training experience and higher levels of upper-body strength. Third, there is some evidence that in cycle ergometry, for determination of a linear power output- $\dot{V}$ O_2_ relationship, it is important to choose a fixed pedaling frequency (between 60 rpm and 100 rpm) and to maintain this pedaling frequency during the exhausting exercise used to determine the maximal AOD (to avoid variations caused by different pedaling frequencies).^33^ Although these conclusions were drawn from studies focusing on cycle ergometry, the same might also be true for resistance exercise. Specifically, while the lifting tempo was similar between the light-load submaximal sets (used for the plotting the relative load- $\dot{V}$ O_2_ relationship) and the TBT condition, this was not the case for VBT. However, given that the AOD obtained at set failure did not differ between conditions, this methodological aspect does not seem to have exerted any meaningful impact at this particular level.

## Conclusion

The present study demonstrates that a VBT approach (while controlling the eccentric phase) represents an effective strategy to enhance volume load in response to the bench press exercise performed to failure at 70% 1RM. Moreover, this effect occurs in a context that does not compromise time under tension during exercise. Finally, we provide preliminary data showing that volume load enhancement resulting from lifting with a VBT approach is associated with a higher contribution of aerobic energy supply to the bench press exercise. Ultimately, these findings may be of practical interest for strength and conditioning professionals. Given the potential relevance of volume load for muscle hypertrophy,^15^ we contend that benching to failure with a VBT approach might enhance chronic adaptations in muscle mass resulting from resistance training. Further research should be conducted to explore such a relationship.
